# Intrigues of biofilm: A perspective in veterinary medicine

**DOI:** 10.14202/vetworld.2016.12-18

**Published:** 2016-01-02

**Authors:** Umar Faruk Abdullahi, Ephraim Igwenagu, Anas Mu’azu, Sani Aliyu, Maryam Ibrahim Umar

**Affiliations:** 1 Department of Postgraduate, Faculty of Medicine, Universiti Sultan Zainal Abidin, Kuala Terengganu, Malaysia; 2 Department of Veterinary Pathology, University of Maiduguri, Maiduguri, Nigeria; 3 Department of Microbiology, Faculty of Medicine, Universiti Sultan Zainal Abidin, Kuala Terengganu, Malaysia

**Keywords:** antibiotic resistance, biofilm, contact surfaces, quorum sensing, veterinary medicine

## Abstract

Biofilm has a tremendous impact in the field of veterinary medicine, especially the livestock industry, leading to a serious economic loss. Over the years, little attention has been given to biofilm in animals with most of the research geared toward human biofilm diseases. The greatest challenge posed by biofilm is in its incredible ability to resist most of the currently existing antibiotics. This mystery can best be demystified through understanding the mechanism of the quorum sensing which regulate the pathophysiology of biofilm. Ability of biofilm formation in a variety of inanimate surfaces such as animal food contact surfaces is responsible for a host of biofilm diseases affecting animals and humans. In this review, we highlighted some of the challenges of biofilm in livestock and food industries. Also highlighted are; mechanisms of biofilm development, best diagnostic approach and possible novel therapeutic measures needed to combat the menace of biofilm in veterinary medicine.

## Introduction

Biofilm-associated diseases pose serious health challenges to the animal kingdom, resulting in high economic losses in the livestock industry [1,2]. It is suggested to be responsible for about 80% of infectious diseases affecting animal and human, hence the impact in veterinary medicine cannot be ignored. Pathophysiology of the most biofilm infections in animal are similar to that of human, according to previous studies, approximately 61% of human biofilm infections are of zoonotic origin [3], thus, underscores the importance of biofilm in veterinary medicine and the need to come up with a robust treatment and preventive plan toward combating the scourge. Few examples of common zoonotic biofilm infections include; chronic wound resulting from dog bite injury. The increased chronicity of wound following a dog bite injury is connected to the pathogenic effect of numerous biofilm organisms in the oral cavity of the dog [4]. Another example is canine uropathogenic *Escherichia coli* infection which affects the human urinary system; this bacterial biofilm has been experimentally demonstrated to induce cytotoxicity in human bladder epithelial cells [5]. Other common zoonotic biofilm is highlighted in the list of animal biofilm diseases (Table-1). In this review, we highlighted some of the challenges of biofilm in livestock and food industries. Also highlighted are; mechanisms of biofilm development, best diagnostic approach and possible novel therapeutic measures needed to combat the menace of biofilm in veterinary medicine.

**Table-1:** Common biofilm diseases in veterinary medicine.

S/N	Diseases	Common aetiology	Most affected host	Reference
1	Mastitis	*Streptococcus Agalactiae* & *Staphylococcus aureus*	Domestic ruminants	[67-69]
2	Jones disease	*Mycobacterium avium sub specie paratuberculosis*	Small ruminants	[70,71]
3	Pnumonia	*Pasturella multicida*	Avian and ruminants	[72]
4	Caseous Lymphadinitis	*Coryenobacterium pseudotuberculosis*	Small ruminants	[73]
5	Liver abscess	*Fusobacterium necrophorum*	Domestic ruminant	[74]
6	Wound infection	*Staphylococcus aureus* & *Pseudomonas* spp	Equine	[67,68,75,76]
7	Enteritis	*Escherichia coli* & *Salmonella* spp	All domestic animals	[1]
8	Urinary tract infection	*Escherichia coli*	Dogs	[77]
9	Pyometra	*Escherichia coli*	Dogs	[78]
10	Periodontal disease	*Staphylococcus* spp	Dogs & Cats	[4]

## Development of Biofilm

**Figure-1:**
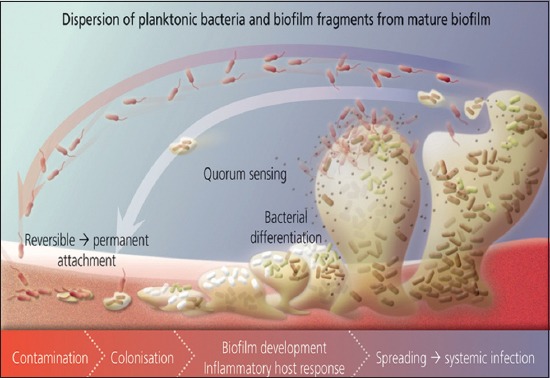
Sourced from Wikimedia: Schematic representation of polymicrobial biofilm development.

Biofilm formation is a complex process, occurring as a cascade of molecular and physiological events. It can be classified into five distinct stages, which includes: (1) Development of a surface conditioning film, (2) reversible and irreversible attachment of cells to a surface, (3) formation of microcolonies, (4) maturation and differentiation of the biofilm with the expression of matrix polymers, and (5) dispersal of cells from the biofilm [6-8]. The mechanism of biofilm formation (Figure-1).

## Development of a Surface Conditioning Film

Surface conditioning film is a complex surface consisting of polysaccharides, glycoproteins, and humic compounds [9-11]. It serves as the platform for surface adherence of microorganism, hence, seen as pre-requisite to the attachment stage. The conditioning film playsa key role in modifying the physical and chemical properties of the substratum, as well as providing a concentrated nutrient source and important trace elements [11]. However, components of blood, tears, urine, saliva, intravascular fluid and respiratory secretions in an animal can also contribute to conditioning film. Within the natural environment, microorganism does not adhere directly to a substratum, rather, they adhere to a conditioning film which is known to form on much substrata, Figure-1 demonstrates the role of conditioning film in biofilm development [11,12].

### Adhesion/attachment

This is a crucial stage in biofilm formation involving attachment or adhesion of microorganism, the process occurs in two steps, reversible and irreversible adhesion [11,12]. Reversible adhesion (attachment) is an initial weak attachment of microbial cells to a surface while irreversible adhesion occurs as a permanent bonding of microorganisms to a surface. Attachment occurs reversibly due to the impact of either hydrodynamic or repulsive forces; it can also be as a result of a response to the absence of nutrient availability [12-15]. Cellular and extracellular appendages play a vital role in microbial surface attachment. The common appendages often used in bacterial attachment to plant and animal tissues surfaces include pili and fimbriae, which consist of multiple different appendages, and other structures such as curli, adhesins, intimins, and invasins [16-19].

Attachment of bacteria has been found to stimulate expression of numerous genes that activate release of extracellular matrix and subsequent development of biofilm [11,20]. Thus, biofilm formation progresses when bacteria adhere to surfaces in aqueous environment and begin to excrete a slimy glue-like substance that can anchor them to a variety of materials including, metals, plastics, soil particles, medical implant materials and most significantly animal or human tissue [21], Figure-1 illustrates how biofilm attachment takes place.

## Growth and Development/Microcolonies Formation

Microbial growth, development of microcolonies and recruitment of additional microorganisms occurs after adsorption of macromolecules and attachment of microbial cells to a substratum. Subsequently, after the initial colonization, the biofilm grows through a combination of cell division and recruitment. Microcolonies are three to five layers deep community of bacterial cell [22]. As the attachment of microorganism occurs, the colonizing bacteria grow with the production and accumulation of extracellular polymers. Consequently, the cells are dependent on substrate flux from the liquid phase and/or exchange of nutrient with neighboring cells in the biofilm [11]. It is important to note that mere attachment of microorganism to a surface does not imply the formation of microcolony, hence, a coherent cell to cell interactions are needed to establish and hold the microcolony together [23].

### Detachment/dispersal

Detachment of cells from the biofilm colony and their dispersal into the environment marks the final and indeed an essential stage of biofilm life cycle; this contributes to biological dispersal, bacterial survival, and disease transmission. Like other stages of biofilm development, dispersal can be a complex process involving numerous environmental signals, signal transduction, pathways and effectors [24]. To date, detachment remains poorly researched and understood, which therefore, complicates the formation of satisfactory models [11]. Biofilm detachment can also occur as a result of a low nutrient condition indicating a homeostatic mechanism, which may be genetically determined. Therefore, detachment is not just important for promoting genetic diversity, but also escaping unfavorable habitat aiding in the development of new niches [11].

Thus, once biofilms are established planktonic bacteria may periodically leave biofilm on their own and when they do, they can rapidly multiply and disperse. The dispersal or shedding of planktonic cells from a biofilm may be essential to permit bacteria escape from confines of the biofilm in order to colonize new locations [1].

Bacterial dispersal can be divided into three distinct phases; (i) Detachment of cells from the biofilm colony, (ii) Translocation of the cells to new locations, (iii) Attachment of cells to a substrate in the new location [25]. In general, mechanisms of biofilm dispersal can be divided into two broad categories which are; active and passive dispersal. Active dispersal refers to mechanisms that are initiated by the bacteria themselves, whereas passive dispersal refers to biofilm cell detachment that is mediated by external forces such as fluid shear, abrasion (collision of solid particles with the biofilm), predator grazing, animal and human intervention [26,27], Figure-1 illustrates how dispersal pattern of biofilm and planktonic bacteria are exhibited.

## Microbial Food Contact Surface in Veterinary Medicine

Under favorable conditions, an array of existing spoilage and pathogenic microorganisms frequently comes in contact with various surfaces within animal habitats. This consequently, poses a serious challenge in the animal and human food industry. Common microbial contact surfaces in the livestock industry includes, animal feeding troughs, drinkers, and other routinely used glass, plastic and polypropylene utensils [28,29]. Thus, contact of spoilage and pathogenic microbes with these surfaces can result in biofilm formation, causing spoilage of food as well as various pathological conditions in animal [28-30].

The most common microbial food contact surfaces leading to food spoilage are *Pseudomonas fragi*, *Pseudomonas aeruginosa*, *Micrococcus* spp. and *Enterococcus faecium* while the likes of *Listeria monocytogenes*, *Yersinia enterocolitica*, *Staphylococcus aureus*, *Salmonella typhimurium* and *E. coli*, belongs to the pathogenic class [31-33]. Biofilm has also been found in several contact surfaces available in diary plants. Microbes like, *Streptococcus* spp., *Shigella* spp. and *E. coli* often comes in contact either directly with the animals or milking equipment, resulting in the development of biofilm [34,35]. However, this causes contamination of the dairy products and diseases among affected animals, as well as serving as potential zoonosis [36,37].

## Biofilm Antimicrobial Resistance in Veterinary Medicine

The ability of bacterial pathogens to resist adverse conditions, while inflicting damage on its host depends on the capacity to form biofilm [2]. Biofilm are much more resistant to antimicrobial agents when compared to free-flowing planktonic bacteria, in most cases, over a thousand times more concentration of an antibiotics required to kill planktonic bacteria will be needed to destroy biofilm bacteria [1,38]. Multi species biofilm are more resistant to antimicrobial agents, although some genetically heterozygous single species biofilms are highly resistant to antimicrobial therapy as well as the host immune response. Restriction of antimicrobial agents from gaining access to the pathogens due to the impervious nature of the biofilm encased extracellular matrix, plays a pivotal role in biofilm antimicrobial resistance [39].

Strong protection is conferred on biofilm organisms by the encapsulating self-produced extracellular polymeric substances, this substances keeps biofilm extracellular enzymes in proximity to the cells [2,40]. Enzymes produced by biofilm polymeric substances plays a key role in protecting the bacteria organism through metabolizing of biopolymers and other substances used as antimicrobial agents [40,41]. Common extracellular enzymes produce by the biofilm polymeric substance are aminoglycoside modifying enzymes (AMEs) and beta-lactamase [42,43]. This property is posed by *Acitenobacter baumannii*, a short and rod-shaped Gram-negative bacterium belonging to the “ESKAPE” group of pathogens known for causing serious nosocomial infection among hospitalized animals and humans [44]. High aminoglycoside resistivity exhibited by these pathogens is mainly due to AME produced by the bacterial biofilm [42,45]. As revealed in various researches, animal and human pathogenic bacteria that produces beta-lactamase enzyme are referred to as beta-lactam producing bacteria (BLPB) [46]. These classes of bacterial organisms are responsible for a number of antimicrobial resistant biofilm infections in veterinary medicine [43,46]. Example of animal derived BLPB are *Salmonella* spp., *E. coli*, *Enerococcus* spp., *Campylobacter* spp., and *Staphylococcus* spp among others [43]. This enzyme inhibits the antimicrobial actions of beta-lactam antibiotics, as demonstrated in the inhibitory action of beta-lactamase to penicillin antibiotic [47].

The role of BLPB in promoting biofilm resistance against beta-lactam among non-BLPB pathogens have been demonstrated in an *in vitro* study showing resistance of non-BLPB to penicillin owing to the release of beta-lactamase enzyme within the environment [47], perhaps this throws more light on the concept of multi species biofilm antimicrobial resistivity. Recently, another common case of biofilm antimicrobial resistance was demonstrated in a study, revealing the development of *E. coli* biofilm in canine urinary tract infection and resistance of the condition to fluroquinolones therapy [48]. According to a recent study conducted on the detection of virulence among strains of coagulase negative *Streptococcus* (CNS), reveals a strong connection between presence of certain genes that promotes virulence among the highly pathogeneic strains of CNS biofilm [49]. Similarly, in two separate studies, virulence genes (*hlyA, plcA, actA*, and *iap*) responsible for pathogenicity of listeria monocytogens was identified in milk of goats, sheep and camels which potentially poses a great zoonotic threat to human, when such dairy products are consumed [50,51]. Hence, understanding the genetic mechanism of biofilm development would be of great importance in interfering with formation of biofilm. Generally, biofilm resistance to antimicrobials explains the puzzling nature of urinary tract infection in both animals and humans, occurring mostly as a recurrent infection [48,52].

## Diagnostic and Therapeutic Approach of Biofilm Infection in Veterinary Medicine

As a rule of thumb, effective treatment of any disease requires accurate diagnosis of the disease [53]. However, due to the complex nature of biofilm, achieving accurate diagnosis through the conventional culture and isolation diagnostic method is quite difficult [53,54]. Adherence of microbes to specific parts of an animal host often results in negative culture result, in some cases false positive result is obtained due to the presence of free moving planktonic bacteria. Therefore, diagnostic approach tailored toward identifying specific microbes will be most appropriate for biofilm diagnosis.

At present, diagnostic techniques such as; serology, fluorescent *in situ* hybridization, conventional radiographic approaches (computed tomography, magnetic resonance imaging and radioinuclide scans), polymerase chain reaction, loop-mediated isothermal amplification and other molecular technique has shown promising result in effectively diagnosing biofilm diseases [53-57]. Use of non-invasive microscopic imaging technique known as laser scanning confocal microendoscopy has been successfully used in diagnosis of mucosal biofilm infection. This laser scanning method promotes easy observation of biofilm in mucosal biofilm of hollow organs such as the lower gastrointestinal tract, middle ear and the urinary tract [56]. Use of specific biofilmvirulence gene markers, will not only be a useful tool of identifying biofilm but exposes the presence of certain virulent biofilm in clinical samples, a good example is identification of the genes in milk of cows suffering from mastitis [50,51].

Effective treatment of biofilm infection requires dual approach through combination of antibiofilm and antimicrobial agent [58]. The pathophysiology of biofilm infection is thought to be regulated by the quorum sensing mechanism, through a cascade of event in which community of microorganism, united as a single entity expresses gene virulence, and the antimicrobial properties [59-61]. Proper understanding of the concept of quorum sensing phenomenon is key toward developing an effective means of combating biofilm. Conventional antimicrobial approach has a restricted range of action against fast growing pathogenic organism with little or no effect on biofilm [62]. However, more radical therapeutic approach, involving the combination of conventional antimicrobial with devices like:

### Ultrasound

This device enhances the bacteriocidal action of the antimicrobial agent, through passage of non-invasive acoustic energy waves through the skin to the site of biofilm [62]. Ultrasonic energy is also used to enhance the release of drug from delivery devices, and elicits antimicrobial action by promoting biofilm cellular membrane destruction, hence enables active or passive uptake of antibiotics [62].

### Electric current

Synergetic use of low level electric current with antibiotics enhances the antimicrobial activity of antibiotic which ordinarily are resisted to by biofilm organism(s) [62-64]. The electromagnetic pulse will increase the antimicrobial activity of cationic antibiotics against bacterial biofilm, example is the simultaneous release of electromagnetic impulse with administration of gentamicin against *S. aureus* [62].

### Phage therapy

This is a robust therapeutic approach, although not commonly employed in veterinary biofilm therapeutics, it however, involves the use of protein that encapsulates DNA or RNA genome to elicit strong bacteriocidal actions at the site of a biofilm infection. The mechanism through which phage achieves its antibiofilm action is by enzyme production which hydrolyses and degrades the extracellular matrix of biofilm, perhaps, the use of bacteriophage or combination with antibiotic will be effective [65,66].

### Drug delivery system

This system involves combination of antimicrobial drugs with nano-carriers. Antimicrobials such as gentamycin, ampicilin, ciprofloxacin among others are encapsulated in a drug delivery nano-carrier. Examples of commonly used nano-carriers include phosphotydyl-choline, polyethylene glycerol, polyamidoamine, and polyacrylate. Mechanism of action of nano-carrier is basically through prolonging the action of the active molecules which is been delivered to the appropriate action site, this approach has proved effective against biofilm [62].

## Conclusions

Additional research is needed to unravel the mystery of biofilm. Present trend of biofilm in veterinary medicine suggest persistence of animal and human health challenges in the future, leading to greater economic loss. Strict adherence to aseptic practice in the livestock industry will go a long way in stemming the menace of biofilm. Control of illicit and indiscriminate use of antibiotics, will also help to address some of the challenges posed by biofilm antibiotic resistance. Application of novel therapeutic approach such as, phage therapy and the use of some mucolytic agents that is capable of inhibiting biofilm formation, are highly recommended. Effort should be targeted at interfering with development of biofilm rather than focusing on treatment which is often difficult to achieve, this can be realized through improved studies on the genetic mechanism of biofilm development. Furthermore, combination of antibiotics with certain devices like the nano-carriers, ultrasound and other recent technology, which uses controlled level of electric current in promoting antibacterial activity of antibiotics has shown promising result.

## Authors’ Contributions

UFA, EI and AM conceived the idea and design. Assembling of resource was done by UFA, AM, and SA. All the authors contributed in the drafting and editing of the manuscript. All authors read and approved the final manuscript.
